# The added value of haemoglobin to height, age, and sex to predict DLCO in subjects with preserved exercise capacity

**DOI:** 10.1371/journal.pone.0289540

**Published:** 2023-08-08

**Authors:** Eldar Priel, Nermin Diab, Matthew Patel, Mustafaa Wahab, Andreas Freitag, Paul M. O’Byrne, Kieran J. Killian, Imran Satia

**Affiliations:** 1 Department of Medicine, McMaster University, Hamilton, Canada; 2 Firestone Institute for Respiratory Health, St Joseph’s Healthcare, Hamilton, Canada; Muhimbili University of Health and Allied Sciences School of Medicine, UNITED REPUBLIC OF TANZANIA

## Abstract

**Background:**

The single breath diffusion capacity for carbon monoxide (DLCO) captures several aspects of the role of the lung in meeting the metabolic demands of the body. The magnitude of the independent contributors to the DLCO is unknown. The aim of this study was to investigate the factors that independently contribute to the DLCO.

**Objectives:**

The objective was to investigate the impact of height, age, sex and haemoglobin on DLCO, alveolar volume (VA) and carbon monoxide transfer coefficient (KCO).

**Methods:**

Study participants were pre-screened based on normal exercise capacity achieved during an incremental cardio-pulmonary exercise testing (CPET) using cycle ergometry at McMaster University Medical Center between 1988–2012. Participants who had an FEV1>80% predicted, with an FEV1/FVC ≥0.7 and who achieved a maximum power output ≥80% were selected for analysis. In total, 16,298 subjects [61% male, mean height 1.70m (range 1.26–2.07), age 49 yrs (10–94), weight 79 kg (23–190) had DLCO measured while demonstrating normal spirometry and exercise capacity.

**Results:**

The DLCO increased exponentially with height, was 15% greater in males, increased with age yearly until 20, then decreased yearly after the age of 35, and was 6% higher per gram of haemoglobin (5.58*Height(m)^1.69^*1.15 in Males*(1–0.006*Age>35)*(1+0.01*Age<20) *(1+0.06*Hb gm/dl), (r = 0.76).

**Conclusion:**

Height, age, sex, and haemoglobin all have independent influence on the DLCO in subjects with normal spirometry and preserved exercise capacity.

## Introduction

In 1914, Marie Krogh reported that carbon monoxide (CO) is transported at a maximum rate of 20 to 50 ml/mmHg/min from the alveoli to the circulating blood [1]. Based on the physical principles of diffusion, the maximum rates of transport are dependent on the solubility of the gases divided by the square root of its molecular weight and would predict the maximum rate for oxygen transport of 1.23*DLCO, and for carbon dioxide (CO_2_) transport of 24.6*DLCO, i.e., diffusion of CO_2_ was 20-fold greater. Despite this initial emphasis on diffusion, the DLCO is now known to capture communicating alveolar volume (VA), ventilation, perfusion, haemoglobin, carboxyhaemoglobin, blood flow and their interrelationships across individual alveolar units. Hence, capturing a single measurement covering such a broad range has obvious clinical appeal in diagnosis, rating disease severity and responses to treatment. Half a century later, measurement of the DLCO emerged in a clinical context and progressed with the commercial development of simplified testing [2]. The overall aim of this study was to investigate the factors that independently contribute to the DLCO. The effects of height, age and sex are accounted for in most reference equations. However, despite haemoglobin being the only carrier of oxygen and the observations seen in clinical practice of reduced DLCO in patients with anaemia [3], the magnitude of the effect of haemoglobin is unclear. In this study we used cardiopulmonary exercise testing (CPET) to define functional capacity. Participants who had a preserved maximal power output with no spirometric deficit were defined as functionally normal, regardless of diagnostic labels or symptoms. The magnitude of the independent contribution of different components to the DLCO is reported.

## Methods

### Study design and subjects

This was a retrospective study based on data collected from sequential patients referred for clinical exercise testing at McMaster University Medical Center from 1988–2012. The indication for exercise testing was broad with the commonest indications for the assessment of exercise induced chest pain, shortness of breath, fatigue, and rehabilitation purposes. This population includes normal subjects and subjects with the common cardiopulmonary disorders of varying severity. Subjects who failed to exercise were excluded as they included patients unwilling to exercise and those limited by severe musculoskeletal pain. All the CPET testing was conducted in a clinical laboratory setting and informed verbal and written consent was taken from patients, parents or guardians prior to testing for performance of the test and use of this anonymized data for future research purposes. This study was approved by the Hamilton integrated research ethics board (14409-C) as a retrospective chart review.

### Study population

A total of 37,672 subjects performed incremental cycle ergometry to symptom limitation and had a DLCO measured immediately prior to exercise. The total population were explored to ascertain the contribution of height, age, sex, muscle strength, spirometry and DLCO. Normal predicted values relative to height, age and sex were derived from 16,298 (43.2%) who had a normal exercise capacity and normal spirometry (Table 1).

**Table 1:** Demographics and physiological parameters.

	SUBJECTS (n = 16,298)			
Variable	Mean	S.D.	Min	Max
**Male (%)**	61			
**Age**	49.66	16.76	10.00	94.00
**Height (m)**	1.70	0.10	1.26	2.07
**Weight (kg)**	79.94	17.67	23.00	190.60
**BMI**	27.57	5.13	12.31	61.21
**FEV1 (L)**	3.12	0.80	1.00	7.00
**FEV1% Predicted**	100.00	14.58	49.60	159.76
**FVC (L)**	3.81	0.98	1.30	8.00
**FVC % pred**	110.22	17.40	51.64	189.45
**FEV1/FVC (%)**	81.91	5.49	70.00	100.00
**DLCO (ml/mmHg/min)**	24.88	6.36	10.00	54.40
**VA (L)**	5.48	1.27	1.70	10.20
**KCO (ml/mmHg/min/L)**	4.60	0.86	0.48	11.60
**Hb (g/dL)**	13.95	1.33	10.00	21.60

BMI- Body Mass Index; DLCO- diffusion capacity for carbon monoxide; FEV1- Forced Expired Volume over one Second; FVC- Forced Vital Capacity; Hb- Haemoglobin; KCO- The carbon monoxide transfer coefficient; SD–standard deviation; VA- Single breath lung volume.

### Study procedures

After the risks of exercise were explained, informed consent for exercise testing was obtained. Clinical exercise testing done at McMaster University Medical Center includes spirometry relative to time and volume, DLCO (Single breath lung volume[VA] & carbon monoxide transfer coefficient [KCO]), and capillary blood gas [4]. Spirometry was measured with a maximum inspiratory and expiratory maneuver from residual volume (RV) to total lung capacity (TLC) yielding forced vital capacity (FVC) and forced expired volume over one second (FEV1), peak expiratory flow rates (PEFR), forced expired volume at 25, 50 and 75% of the expired vital capacity, peak inspiratory flow rate at FIF 25, 50 and 75%. VA (Single breath lung volume), KCO and DLCO were measured [5]. Haemoglobin (Hb), carboxyhaemoglobin (HbCO), oxygen saturations (SaO2), and arterialized capillary blood gases were measured.

### Statistical analyses

All statistical analysis was performed using Statistica version 13.2. Subjects were considered normal if no exercise limitation was observed (Maximal power output (MPO)≥80% predicted) and baseline spirometry was normal (FEV1/FVC≥0.7 and FEV1≥80% predicted). Descriptive demographic data in the total and normal populations are shown as mean, standard deviation (SD), minimum and maximum. Models are described with the estimated mean, 95% confidence intervals together with the derived Pearson r value for each equation.

Stepwise non-linear interactive modelling was performed with DLCO, VA and KCO as the dependent factor in subjects with no exercise limitation. Height, age<20, age>35, sex, haemoglobin were included as the independent contributing variables [6]. Age was dichotomised for the models for DLCO and VA to <20 and >35 to reflect lung volume growth to full skeletal maturity to age 20 and reduction after the age of 35.

## Results

### Contribution of height, age, sex, and haemoglobin in normal subjects to DLCO, VA and KCO

Since height, age, sex, and haemoglobin are interdependent, a biologically plausible non-linear interactive prediction equation was derived in the normal sub-group: *DLCO = 5*.58*Height(m)^*1*.69^*1.15 in Males*(1–0.006*Age>35)*(1–0.01*Age<20)*(1+0.06*Hb gm/dl), (r = 0.76, Table 2).

**Table 2:** Impact of height, sex, age and haemoglobin on DLCO, VA, KCO.

Variables	Estimate	Lower 95% C.I	Upper 95% C.I.	p-value
**DLCO (ml/mmHg/min):**				
A*Height(m)^b^* C*Males*(1- D*AGE>35)*(1-E*Age<20)* (1+F*Hb gm/dl). r = 0.76.				
**A**	5.577	5.210	5.944	0.000
**B (height)**	1.687	1.625	1.750	0.000
**C (Males)**	0.147	0.138	0.157	0.000
**D (Age>35)**	-0.006	-0.006	-0.006	0.000
**E (Age<20)**	-0.010	-0.011	-0.008	0.000
**F (Hb)**	0.062	0.054	0.070	0.000
**VA (L):**				
A*Height(m)^b^* C*Males*(1- D*AGE>35)*(1-E*Age<20)* (1+F*Hb gm/dl). r = 0.81				
**A**	1.426	1.372	1.480	0.000
**B (height)**	2.250	2.197	2.302	0.000
**C (Males)**	0.149	0.141	0.157	0.000
**D (Age>35)**	-0.001	-0.001	-0.001	0.000
**E (Age<20)**	-0.023	-0.025	-0.022	0.000
**F (Hb)**	0.062	0.054	0.070	0.000
**KCO (ml/mmHg/min/L) :**				
A*Height(m)^b^* C*Males*(1- D*AGE)*(1+F*Hb gm/dl), r = 0.51.				
**A**	5.04	4.77	5.31	0.000
**B (height)**	-0.64	-0.70	-0.59	0.000
**C (Males)**	0.01	0.003	0.018	0.007
**D (Age)**	0.004	0.004	0.004	0.000
**F (Hb)**	0.04	0.037	0.048	0.000

C.I.- Confidence interval; DLCO- diffusion capacity for carbon monoxide; Hb- Haemoglobin; KCO- The carbon monoxide transfer coefficient; VA- Single breath lung volume.

**Fig 1:**
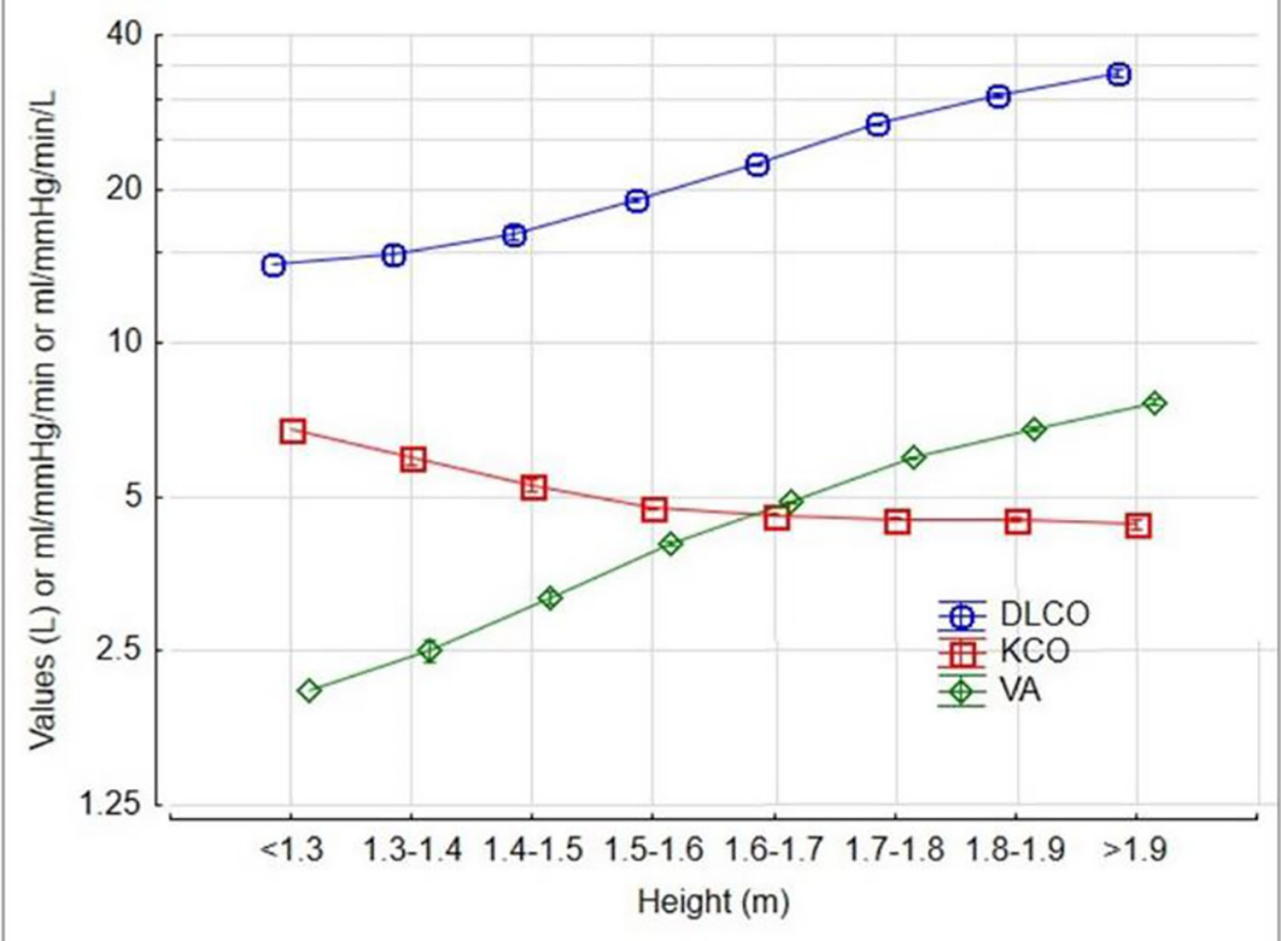
The contribution of height to DLCO, VA and KCO. DLCO = 5.3 *Height^2.9^; VA = 1.0 *Height^3.2^; KCO = 5.7 *Height^-0.4^.

**Fig 2:**
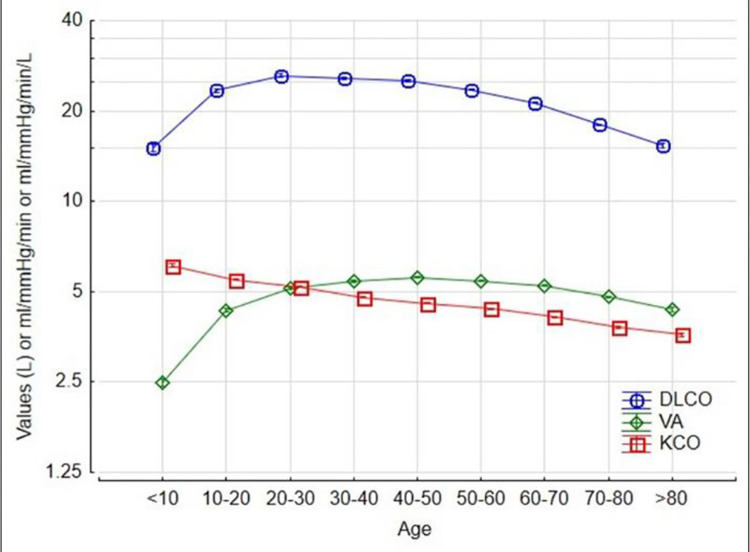
The contribution of age to DLCO, VA and KCO.

**Fig 3:**
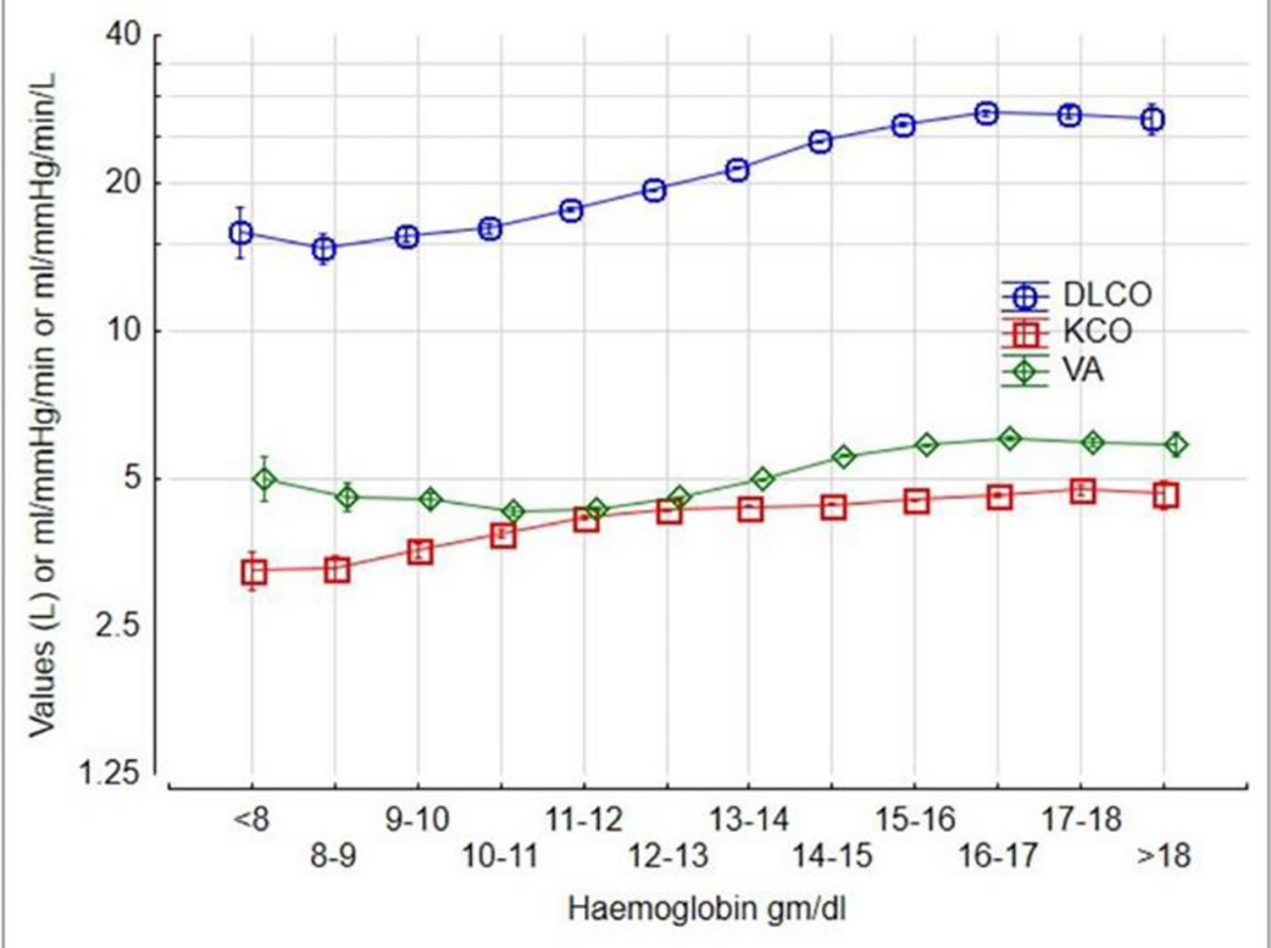
The Contribution of Haemoglobin to DLCO, VA and KCO.

**Fig 4:**
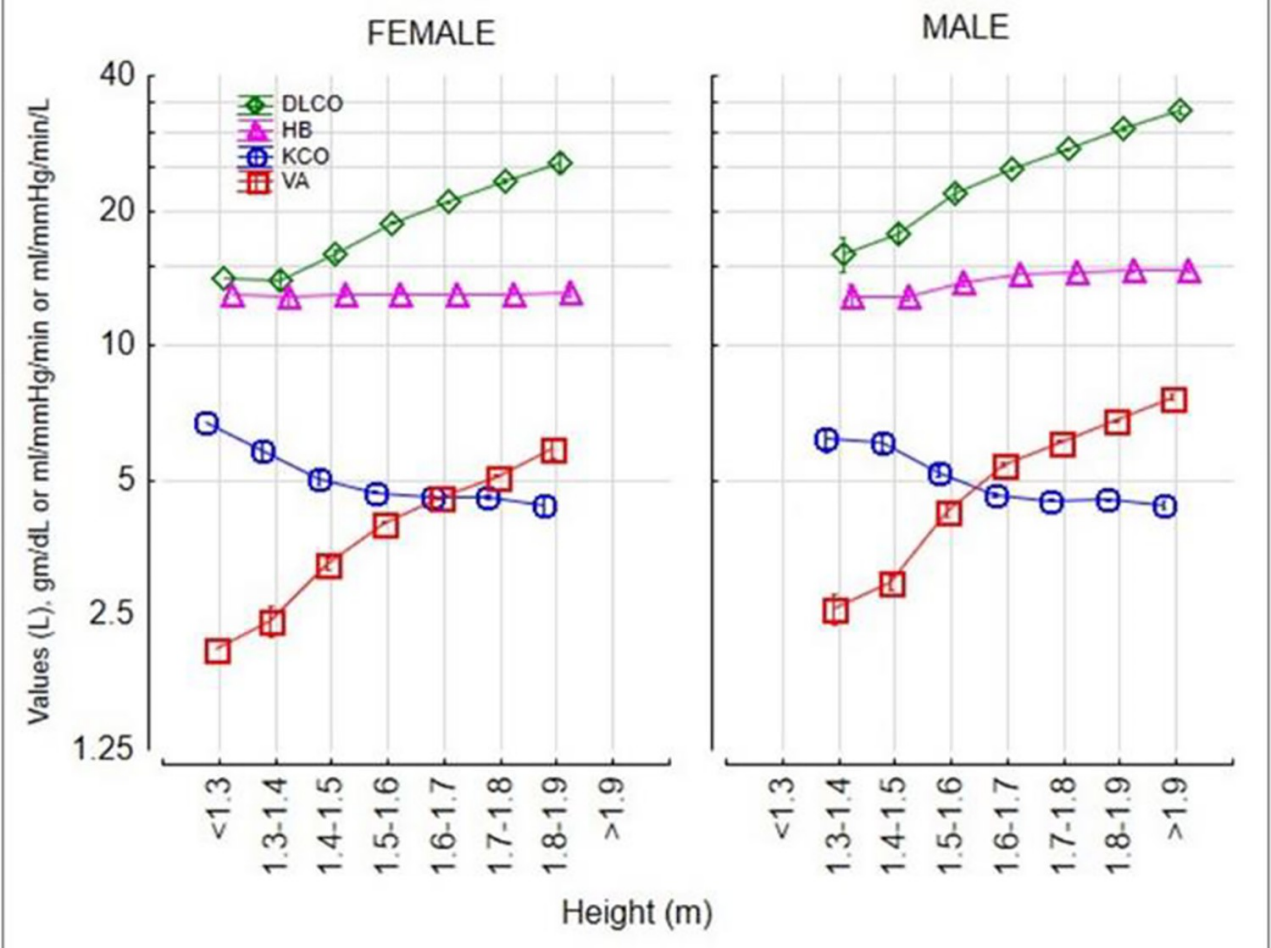
The Contribution of Sex to DLCO, VA and KCO.

The 5th percentile of the DLCO was at 74% predicted. DLCO increased in an accelerating manner with height (Fig 1), gradually increased by 0.1% with age up to 20 at any given height (Fig 2) and then declined by 0.6% per year over the age of 35 (Fig 2), increased by 6% for each gram of haemoglobin increase (Fig 3) and was 15% higher in males (Fig 4). The pattern was the same for alveolar volume (VA) (Table 2, Figs 1–4).

The pattern for KCO was different. The KCO predicted was:

*KCO (ml/mmHg/min/L) = 5*.04*Height(m)^*-0*.64^*1.01 in Males*(1–0.004*Age) *(1+0.04*Hb gm/dl), (r = 0.51, Table 2).

The KCO decreased with height (Fig 1), declined monotonically by 0.4% per year (Fig 2), increased by 4% for each gram of Hb increase (Fig 3), and was 1% higher in males (Fig 4).

## Discussion

Our data demonstrates that the DLCO was 15% higher in males and increased by 6% or 2.1 ml/mmHg/min with each gram of Hb. Age less than 20 and greater than 35 has less than a 1% effect per year, independent of height, sex and Hb. The KCO declines as lung volume increases commonly attributed to a decrease in ventilation perfusion matching with increasing lung volume.

Previous studies have tried to address normality in different populations, generating different reference values based on anthropomorphic features [7–10]. Once a normal range was defined, values below this are interpreted as an impairment in gas transfer capacity. However, an impairment is only as important as the disability it causes. The effort required to perform a certain physical challenge is not different between different ethnicities. Traversing a flight of stairs poses the same challenge to Caucasians, African Americans, and people of Asian ethnicity. This study is unique in that “normality” was determined as subjects with an objectively demonstrated normal capacity to exercise with no spirometric deficits. This is a functional definition based on disability. No random collection of normal subjects based on inclusion and exclusion criteria have ever validated that the subjects included had an objective demonstration of a normal capacity to exercise and were asymptomatic based on the achievement of a normal maximal power with a demonstrated normal symptom response. This novel approach to normal was never used before to our knowledge. In this context, any deficiency in haemoglobin, while influenced results, did not cause functional impairment. These results therefore show the spectrum of normal relationships between the different measured variables and the DLCO.

This study was not designed to provide novel reference equations for this functionally normal population, but to show the additional benefit of haemoglobin, a mostly unmeasured significant component of the DLCO, to age sex and height. When compared to recent studies, some of which were designed to provide regression equations for DLCO [11] our study adds in that quantification of the contribution of hemoglobin becomes available.

The DLCO, initially introduced to measure diffusion capacity, is dependent on ventilation /perfusion distribution across alveoli, ventilation and perfusion relative to alveolar volume distribution, ventilation, diffusion and perfusion distribution across the lungs, Hb, cardiac output and any variations in the chemical reactions and ion shifts required for oxygen carriage. In this sense, the DLCO is a very useful measurement of gas exchange. The utility of the DLCO is under appreciated relative to spirometry. The DLCO is determined by the product of two separate measurements, VA and KCO, and each are influenced by different factors [12]. The VA- single breath lung volume, alveolar volume, communicating lung volume or accessible lung volume, is influenced by the number and expansion of the alveoli and in turn alveolar damage. While sex, age, and height influence VA, these result from biological processes. There are known differences in lung development between males and females which starts at an early age [13], such as differences in alveolar size and number contributing to a higher VA in males [14]. The KCO represents the integrity of the capillary bed and the cardiac output. Lower DLCO is associated with higher ventilatory demand during exercise [15] and lower cardiac output [16]. DLCO is currently not tested routinely for patients with unexplained dyspnea. Unpublished Data from our group supports the notion that the impact of an absolute reduced DLCO in a symptomatic patient warrants investigations to delineate the underlying cause.

In this study, lower Hb was associated with a lower DLCO and KCO. This was expected given that gas exchange across the alveolar-capillary interface is influenced by the concentration of haemoglobin [12, 17]. The relationship between Hb and DLCO was sigmoidal (Fig 3). This could be explained by a higher cardiac output in subjects with a lower Hb, as a compensation for anemia, and relative saturation of Hb at the higher levels. An unexpected finding was that a higher Hb influences VA. As a similar association appears with height over 1.5m and an increased slope until the age of 20, a possible explanation could be the effects of testosterone on erythropoiesis [18].

This is the first study to provide empirical data of Hb, as most pulmonary function testing does not include measuring Hb, including the GLI reference values [19]. The 2005 ATS/ERS standardization document recommends DLCO values should be corrected for Hb using the equation derived by John Cotes and colleagues, which assumes a partial pressure of oxygen of 100mmHg, and a fixed diffusion capacity of 0.7 ml/min/mmHg [17, 20]. These provide a simple linear imputation relative to 14.6g/dL for males over 15 yrs and 13.4 g/dL for females and children, but empirical data from our dataset and from general population were significantly different from these [21, 22]. This study found it to be the third most important contributor to DLCO and supports the ATS/ERS recommendations to correct measurements to an individual’s Hb level [23, 24].

There are limitations to this study. First, this is a retrospective study in a single centre potentially leading to selection bias. However, this can also be advantageous as it reduces errors between sites using different equipment and performed at different altitudes. Second, the reference values were not calculated from healthy controls per se, from the general community, but in patients with a normal exercise test and normal spirometry. Random selection from the general community could have been used, but this would not guarantee a ‘healthy control’. Third, we did not address the representation of the data as a percentage of predicted normal values because the physiological costs are absolute and not relative to a predicted mean. Fourth, our criteria for normality (i.e., FEV1/FVC ratio, normal FEV1, MPO>80% predicted) may be a source of selection bias, since the FEV1/FVC ratio is age dependent. We believe that the small numbers of patients in the extremity of ages, the minimal influence of age on the DLCO and the large overall number of study participants, together with normal functional outcomes, balances any significant bias, however this can not be tested with the available data. Fifth, we do not have data on current or previous smoking or pack year history, which may be relevant information, since smoking can have an effect on DLCO.

## Conclusions

The most important contributors to DLCO in a population with no exercise impairment and normal spirometry are height, sex, Hb and age. Haemoglobin is an important contributor to DLCO, and should be incorporated into measurements when performing pulmonary function testing.

## Abbreviations

CPET: cardiopulmonary exercise test
CI: Confidence interval
DLCO: diffusion capacity for carbon monoxide
FEV1: Forced Expired Volume over one Second
FIF: Forced Inspiratory Flow
FVC: Forced Vital Capacity
Hb: Haemoglobin
KCO: The carbon monoxide transfer coefficient
MPO: maximal power output
PEFR: Peak Expiratory Flow Rate
SaO2: Oxygen Saturation
SD: standard deviation
TLC: Total Lung Capacity
VA: Single breath lung volume
